# Supplementary material for: Propagating bands of plastic deformation in a metal alloy as critical avalanches
Source: Sci Adv. 2020 Oct 7;6(41):eabc7350. doi: 10.1126/sciadv.abc7350 (PMC7541064; doi:10.1126/sciadv.abc7350)
Supplement: abc7350_SM.pdf [file abc7350_SM.pdf]

Science Advances
AAAS

advances.sciencemag.org/cgi/content/full/6/41/eabc7350/DC1

Supplementary Materials for

Propagating bands of plastic deformation in a metal alloy as critical avalanches

Tero Mäkinen*, Pasi Karppinen, Markus Ovaska, Lasse Laurson, Mikko J. Alava

*Corresponding author. Email: tero.j.makinen@aalto.fi

Published 7 October 2020, *Sci. Adv.* **6**, eabc7350 (2020)
DOI: 10.1126/sciadv.abc7350

The PDF file includes:

Legend for movie S1

Other Supplementary Material for this manuscript includes the following:

(available at advances.sciencemag.org/cgi/content/full/6/41/eabc7350/DC1)

Movie S1

Supplementary materials

Supplementary Video 1: An example video of speckle images from an experiment showing the complex dynamics of the deformation bands from the yielding of the sample until the final failure. The color corresponds to the speckle image intensity (from dark to light) which corresponds to the local strain rate.
